# The Development and Application of Tritium-Labeled Compounds in Biomedical Research

**DOI:** 10.3390/molecules29174109

**Published:** 2024-08-29

**Authors:** Yu Teng, Hong Yang, Yulin Tian

**Affiliations:** State Key Laboratory of Bioactive Substances and Function of Natural Medicine, Beijing Key Laboratory of Active Substances Discovery and Drugability Evaluation, Institute of Materia Medica, Peking Union Medical College and Chinese Academy of Medical Sciences, Beijing 100050, China; 15041498582@sina.cn

**Keywords:** tritium labeling, synthesis of tritiated compounds, protein binding studies, autoradiography

## Abstract

With low background radiation, tritiate compounds exclusively emit intense beta particles without structural changes. This makes them a useful tool in the drug discovery arsenal. Thanks to the recent rapid progress in tritium chemistry, the preparation and analysis of tritium-labeled compounds are now much easier, simpler, and cheaper. Pharmacokinetics, autoradiography, and protein binding studies have been much more efficient with the employment of tritium-labeled compounds. This review provides a comprehensive overview of tritium-labeled compounds regarding their properties, synthesis strategies, and applications.

## 1. Introduction

Radionuclide-labeled compounds have been widely used in a vast number of studies, especially in the life sciences [1,2,3,4]. Radionuclides are attractive in drug development as they exclusively emit intense signals without structural changes to the drugs. The high specific activity of molecules labeled with tritium is easy to visualize, track, and quantify, which makes them powerful tools for pharmaceutical studies. In addition to the drug metabolism and pharmacokinetics studies, tritium-labeled compounds are widely employed in other research areas such as receptor binding and autoradiography [5,6,7,8,9,10].

The International System of Units quantifies the radioactivity of tritium-labeled compounds in terms of the Becquerel (Bq), whereas the Curie (Ci) is the traditional unit of measure [11]. Another important parameter in radionuclide chemistry is specific activity (SA), which is expressed in terms of radioactivity per molar unit. It is the level of SA that determines the application of radionuclides. The SA of tritium is much higher than that of carbon-14 [12], which enables tritium-labeled compounds to be employed in the studies of autoradiography, protein binding, and permeability assays.

The main method to detect the emission of tritium is liquid scintillation counting (LSC). The beta particle emitted by tritium can excite the fluor, which then emits light when relaxing to the ground state [13]. The light detected by the photomultiplier tube is directly related to the amount of radioactivity. A higher sensitivity can be obtained by solid scintillation. Techniques that utilize solid scintillation after automating fraction collection provide better approaches to online radioactivity detection. A higher radioactivity can even be detected by accelerator mass spectrometry techniques, which have a sensitivity of about 1000-fold that of LSC [14]. This technique, however, is limited by the high price of the equipment and the complexity of sample preparation.

One of the important reasons that labeling with tritium is preferred over other radionuclides is its faster, simpler, and cheaper synthesis approaches. On one hand, many classic approaches to labeling, such as tritiodehalogenation, tritiated methylation, and hydride reduction, have experienced a great improvement [15,16,17]. On the other hand, ortho-exchange chemistry has brought revolutionary developments to tritium chemistry [18,19]. Catalyzed by an appropriate metal, hydrogen in the compound can undergo an exchange with a tritium source, which is usually tritium gas or tritiated water.

This review gives an overview of tritium-labeled compounds in three areas. We will first discuss the physical, chemical, and biological properties of tritium-labeled compounds as well as their storage. Then, we introduce the strategies for incorporating tritium labeling. Finally, we discuss the application of tritium-labeled compounds.

## 2. Properties of Tritium-Labeled Compounds

### 2.1. Physical and Chemical Properties of Tritium

Some important properties of tritium in comparison with carbon-14 are listed in Table 1. Thanks to the short range of beta particles emitted by tritium, high-resolution autoradiography and autoradioluminography can be achieved, enabling autoradiographic studies at cellular and subcellular levels [20]. The high specific activity of tritium (28.8 Ci/mmol) is approximately 500 times greater than what can be achieved with carbon-14 (62.4 mCi/mmol) labeling. This makes tritium irreplaceable in macromolecular labeling at low molar concentrations, especially for biomolecules such as peptides, proteins, oligonucleotides, and antibodies. The energy of the beta particle emitted by tritium is much lower than that emitted by carbon-14. It can penetrate the air by only about 5 mm, which is safe enough for workers to manipulate it without special shielding [21]. However, internal exposure to tritium should be avoided. Tritium-labeled compounds must not be ingested or inhaled and must not make direct contact with open wounds. Therefore, wearing suitable gloves and working in fume hoods or glove boxes is required.

Tritium-NMR is a useful tool for the detection and analysis of tritiated compounds [22,23]. As tritium has almost the same chemical shifts as protium, tritium-NMR is widely used to assign the unambiguous site of the tritium label. In addition, diverse 2D-NMR techniques have been employed to analyze complex tritium-NMR spectra [24,25].

Different chromatographic behaviors have been observed between tritiated compounds and their unlabeled counterparts [26]. The shorter and stronger bonds of tritium and carbon result in a change in lipophilicity, which may explain the difference in chromatographic separation [27]. For compounds with high-tritiated labeling, isotopic fractionation is much clearer than that of single-labeled compounds, which are often neglected [28]. It is worth noting that tritiated compounds usually elute ahead of unlabeled compounds through reversed-phase high-performance liquid chromatography.

### 2.2. Storage of Tritiated Compounds

In addition to chemical degradation, the beta particle emitted by the tritium label continuously accelerates the decomposition of tritiated compounds, of which there are three types: internal, external, and secondary decomposition [29]. The first is caused by isotopic decay and accounts for about 5% of tritium decomposition per year [30]. The second is caused by the direct interaction with the high-energy beta particle. The emission energy of beta particles from tritium is about two to four orders of magnitude greater than the average energy of an organic bond. Hence, these bonds are quite vulnerable, especially in the solid or high-concentration liquid state. In addition to the drug molecules, the emitted beta particle can interact with other nearby solvent or air molecules, which are then activated [29]. These activated molecules can then cause secondary decomposition of drug molecules. This approach accounts for the primary aim of these decomposition processes.

The vulnerability of the tritium-labeled compounds emphasizes the importance of short and proper storage. A practical method to reduce external decomposition is to disperse tritiated compounds into an appropriate solvent [10]. It is important that the chosen solvent is highly pure, deoxygenated, and chemically compatible with the compounds. To address the problem of secondary decomposition, solvents are required to absorb and stabilize the energy of beta particles. Benzene and toluene are able to stabilize excited states through their π-orbitals [31] and are therefore often used to dissolve less polar radiochemical compounds. Alcohols such as methanol or ethanol acting as radical scavengers are widely used as solvents or as cosolvents of more polar compounds. On the other hand, solvents like chloroform, dichloromethane, ether, or water are poor choices due to their inclination toward forming radicals [32]. As the decomposition of radioactive compounds is temperature-dependent, it is recommended to store radioactive compounds at temperatures as low as −80 °C, which is usually adequate; however, storage in liquid nitrogen (−140 °C) is optimal [33,34].

### 2.3. Biological Properties of the Tritium Isotope

Potential isotope effects also raise concern in the switching metabolic process of labeled compounds [35]. There are two crucial factors in the kinetic isotope effect: the status of bond hybridization and the type of enzyme involved [36,37]. Many aromatic hydroxylation processes are not likely to show a notable kinetic isotope effect [38]. The rate-determining step of aromatic hydroxylation reactions is an epoxidation reaction through cytochrome p450 oxidation, which does not involve a direct C-H bond breaking [35]. On the other hand, an isotope effect on an aromatic heterocyclic ring was reported to shift the enzymatic oxidation [38]. The scale of isotope effects increases with the number of tritium atoms integrated into the compound. Pharmacological or even safety issues may arise. It is worthwhile to minimize isotope effects and avoid metabolic switching to establish the correct and accurate behavior of a target compound. 

The primary concern about the biological properties of the tritium isotope is the risk of its biological instability. With modern labeling methodology and analytic techniques, loss of the tritium label resulting from chemical and radiochemical processes is no longer a key issue. Tritium loss is mainly due to the introduction of tritium atoms into metabolically vulnerable positions. Furthermore, tritium atom degradation by oxidation or cleavage may even undergo an incorporation with endogenous biomolecules [39,40,41], which would, however, raise the overall radiation in studies involving human beings. In the same molecules, labels at different positions lead to very diverse stabilities [7]. For instance, tritium in the ortho-position of a phenoxy moiety suffers from metabolic instability, while that in the meta position remains quite stable. It is, therefore, of great value to take into detailed consideration the possible metabolic pathways. 

Many metabolically vulnerable positions deserve thoughtful consideration to place a tritium label. N- and O-tritiomethylated compounds are usually uninstable in the metabolic environment, which makes them unsuitable for in vivo studies [42]. The hydroxylation of aromatic systems is mainly through CYP-450-mediated metabolic pathways [43]. The meta or para position of an aromatic system, which often lies on the periphery of the molecule, is not advised when introducing a tritium label. This is worth considering when the tritium labels are incorporated through tritiodehalogenation. The primary hydroxyl group often suffers from hydroxylation by dehydrogenate, so the tritium label adjacent to it is not in an optimal position, while secondary and tertiary hydroxyl tolerates the dehydrogenase pretty well.

## 3. Synthesis of Tritium-Labeled Compounds

### 3.1. Tritium–Halogen Exchange

Great developments in tritium chemistry have been made in the last 30 years [44,45,46], and many improved classical approaches to tritium labeling are also used, such as tritiodehalogenation. With heterogeneous (usually Pd/C) catalysts, tritium can easily take the place of halide in the aryl iodides, aryl bromides, and aryl chlorides.

The main drawback of this approach is the tolerance of reducible groups (other aryl halides, unsaturated bonds, and ArNO_2_), which has been improved in recent decades [47]. By controlling the reaction conditions, tritiodeiodinate can be selectively achieved in the presence of chlorine and even bromine, while tritiodebromination in the presence of chlorine is also possible. A major advantage of this synthetic approach is that the precursor (aryl halides) can be readily synthesized using one-step halogenations. 

**Scheme 1:**
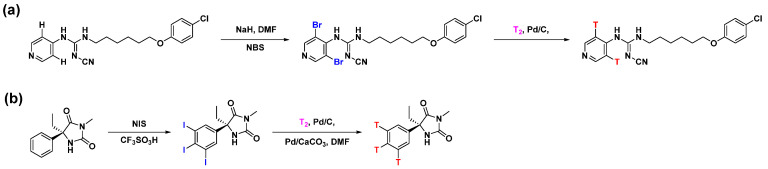
(**a**) Synthesis of tritium-labeled CHS-828; (**b**) synthesis of tritium-labeled (S)-mephenytoin.

Schou and his colleagues successfully detritiated anti-tumor agent CHS-828 in the presence of aryl chlorine with the brominate-then-tritiodebrominate approach [48] (Scheme 1a). The iodinate-then-tritiodeiodinate strategy is a milder alternative to the brominate-then-tritiodebrominate approach [15]. With this approach, Marco and his colleagues prepared the precursor using *N*-iodosuccinimide (NIS) in fluoromethanesulfonic acid and tritiodeiodinate to obtain tritium-labeled mephenytoin (Scheme 1b) [49].

### 3.2. Tritide Reductions

The tritide reduction approach introduces tritium labels through tritium gas or tritium hydrides. Unlike tritium–halogen exchange methods, tritide reductions require a transformation of some precursor chemical. One of the major advantages of catalytic tritiation is that the region and chiral selectivity of tritium labeling can be achieved with a proper catalyst. However, due to the requirement of precursor preparation, this method is infrequently used. 

**Scheme 2:**
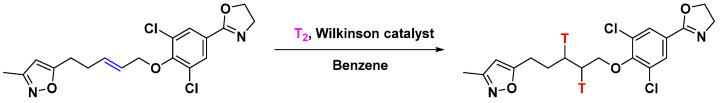
Selective reduction of a double bond without tritiodehalogenation.

Carbon–carbon multiple bonds can be readily reduced with the catalyst in a suitable solvent in the presence of tritium gas, while carbon heteroatom multiple bonds, because of their polar nature, are more favorable for reduction with complex tritides rather than catalytic tritiation. The efficiency of catalytic tritiation is determined by the substrate structure, the activity of the heterogeneous metal catalyst, the support material, and the property of the solvent. Generally, Pd or Rh dispersed on alumina or carbon in protic polar solvents has the greatest ability of reduction, while Ir or Ru dispersed on calcium in aprotic apolar solvents exhibits a much lower reducibility [50,51]. The main drawback of catalytic tritiation is that it can be spread over original carbon–carbon multiple bonds, which is called isotopic scrambling. According to a generally accepted theory, isotope scrambling occurs during catalytic reductions of carbon–carbon multiple bonds through the processes of isomerizations and migrations involving reversible hydrometallations [52,53]. Although tritium–halogen exchange may occur during catalytic tritiation, the selective reduction of unsaturated bonds in the presence of halogens can be accomplished through appropriate selection of the catalyst. Using a Wilkinson catalyst, Egan successfully reduced a double bond without tritiodehalogenation [54] (Scheme 2).

Compared with the tritium gas, it is much easier, using other tritium sources such as LiBT_4_, NaBT_4_, and LiAlT_4_, to introduce tritium in a chemoselective and regioselective manner. Isotope scrambling is also difficult to find in the products of tritide reductions. In addition, higher degrees of stereocontrol can often be achieved by using bulky reagents, chiral tritides, or common achiral tritides in the presence of appropriate chiral catalysts [55].

### 3.3. Methylation

**Scheme 3:**
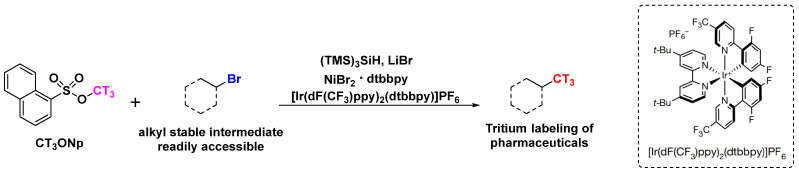
Tritiation of aliphatic pharmaceuticals and radiotracers.

A well-established method for the labeling of drug molecules is the use of several small tritiated building blocks. These building blocks, such as tritiated water, tritiated diimide, tritiated methyl iodide, and tritiated formaldehyde, are of unique utility and commercially available. Especially tritiated methyl iodide and tritiated methyl triflate are the most common building blocks and enable fast and easy reactions with frequently high yields [56,57]. However, N- and O- tritiomethylated compounds are often metabolically unstable, making them unsuitable for many in vivo investigations [58]. In contrast, C-tritiomethylated compounds show much higher metabolic stability, which makes them feasible for in vivo studies as an alternative to tritium labeling. MacMillan et al. [59] reported the radio-methylation of aryl and alkyl bromide catalyzed by metal photoredox and achieved the rapid synthesis of a series of PET radioligands under mild conditions with a wide application range of substrates applied (Scheme 3). This is also the first example of tritium labeling by an alkyl–alkyl cross-coupling strategy to date. 

### 3.4. Metal-Catalyzed Hydrogen Isotope Exchange

Although tritium-labeled compounds can be prepared through chemical synthesis, they may also be prepared directly by exchanging hydrogen atoms with tritium without any change in the chemical structure of the substrates. This method is undoubtedly one of the most dramatic improvements in tritium chemistry [19]. In the presence of an appropriate metal catalyst (usually an Ir or Rh complex), tritium labeling can be achieved with the treatment of tritium gas or tritiated water [60]. Chemoselectivity and regioselectivity can be directed by a wide range of functional groups, most of which tolerate the tritium exchange condition. Compared with the aforementioned method, metal-catalyzed hydrogen-isotope exchange (HIE) labeling has a great advantage in terms of speed and confidence. Regarding these merits, hydrogen isotope exchange has been the method of choice [61]. 

The exchange labeling reactions usually utilize group 8, 9, and 10 metals in tritiated water or mildly acidic solutions. Platinum, palladium, rhodium, ruthenium nickel, and iridium are the most investigated metals for catalytic tritium–hydrogen isotope exchange [62,63]. 

**Scheme 4:**
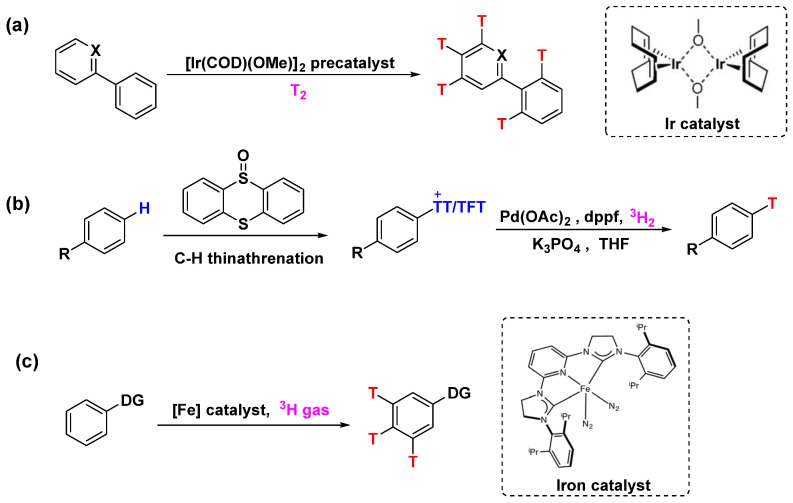
Hydrogen/tritium exchange using an iridium-containing catalyst and a palladium-containing catalyst. (**a**) Multiple site hydrogen isotope labelling of complex molecules using iridium precatalyst [Ir(COD)(OMe)]_2_; (**b**) Chemo- and site-selective C-H tritiation via arylthianthrenium salt by homogeneous palladium catalysis; (**c**) Homogeneous transition Fe-catalysed hydrogen/tritium exchange using tritium gas.

The area of isotopic exchange over homogeneous iridium catalysts has undergone significant expansion since the inception of the technique in the early 1990s [64,65]. These iridium-containing catalysts have the ability to insert into the C-H bond of arenes regioselectivity by directing groups such as ketones, esters, and amides [61]. Along with new iridium-containing catalysts, the activity and selectivity of the iridium complex in the area of tritium labeling have been under detailed investigations, and the overall mechanism of the hydrogen isotope exchange process has been proposed [66,67,68,69]. Pieters et al. [70] reported a general method for the multiple site hydrogen isotope labelling of complex molecules using the commercially available and air-stable iridium precatalyst [Ir(COD)(OMe)]_2_. The multiple site isotope incorporation explained by the in situ formation of a catalytically active system including both monometallic Ir complexes and Ir nanoclusters, and can form complementary catalytic active substances in situ, monometallic iridium complexes, and iridium nanoparticles, which enabled the labeling of a wide range of pharmacologically relevant substructures, including pyridine, pyrazine, indole, carbazole, aniline, oxygen/thiazole, thiophene, and electron-rich phenyl (Scheme 4a). Additionally, many drug molecules on the market, such as Perebron, Celecoxib, and Nilutamide, were labeled with isotopes through hydrogen exchange labeling with iridium-containing catalysts [66]. Recently, HIE reactions using water-soluble Kerr catalysts at different pH solutions have been reported for the first time [71]. The tritium–hydrogen exchange of imidazole, benzoic acid, pyridine n-oxide, tetrazole, and other molecules was realized successfully.

Palladium was found to be generally the most active metal. The C-H tritiation of complex pharmaceuticals with T_2_ gas involving electrophilic C-H palladation offers a novel scope for this tritiation [72]. This practical conversion shows a new substrate range and greater functional group tolerance compared to other tritiation methods, and it has been applied to direct tritiated complex drugs and unprotected dipeptides. Hydrogen hydrolysis catalyzed by palladium is an effective method of molecular tritiation. Ritters et al. [73] reported a well-defined molecular palladium catalyst for homogeneous hydrolysis, with thianthracene as the leaving group for selective drug introduction through late C-H functionalization (Scheme 4b). Unlike the coordination capacity of the traditional leaving group-associated palladium (II) catalyst, an unprecedented dihydrogen catalysis was achieved. This unique reactivity does not require an inert atmosphere or dry conditions, making it practical and stable for having a direct impact on drug discovery and development. Except for single metal catalysis, platinum and palladium catalysts can also be utilized together to achieve synergy efficiency in labeling [74,75].

With the increase in structural complexity of bioactive molecules, it is of great significance to exploit more practical C-H tritiation methods so that valuable tritium radioactive ligands can continue to be used in the investigation of bioactivity. Professor Chirik and collaborators reported an iron-catalyzed method for direct tritium labeling [18]. The regioselectivity of the iron catalyst is orthogonal to that of iridium-containing catalysts (Scheme 4c). This catalyst is tolerated by a range of pharmaceutically relevant functional groups and operates efficiently in polar aprotic solvents at low pressures of tritium gas. 

The efficient and specific introduction of isotopes at specific sites is one of the difficulties of HIE reactions. The usual tritiation conditions often lead to the introduction of multiple isotopes with low regional selectivity. The use of nanoparticles has appeared recently as an attractive solution to overcome this shortcoming. Many types of organometallic nanoparticles (NPs) have been developed to achieve high chemical and regionally selective isotope labeling by adjusting the catalytic activity of nanoparticle size and concentration and the surface ligand properties of metal nanoclusters, or different metals [76,77]. Pieters et al. performed a comprehensive review in this regard [78], so we do not detail it here.

### 3.5. Photoredox-Catalyzed Hydrogen Isotope Exchange

**Scheme 5:**
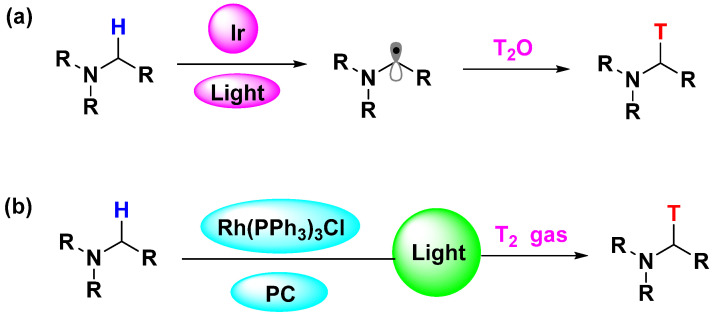
(**a**) Tritiation of HIE through photoredox and HAT catalysis; (**b**) HIE with tritium gas through the merger of photoredox and hydrogenation catalysts.

In addition to the methods mentioned above, there are several strategies that have been developed to achieve a high selectivity of reactions. In recent years, visible light-mediated photoredox catalytic reactions have rapidly developed in organic synthesis. Macmillan first reported in 2017 that photoredox and thiol hydrogen transfer (HAT) synergistic catalytic strategies enable efficient aliphatic hydrocarbon bond isotope exchange (Scheme 5a) [79]. It is worth noting that this strategy can only use tritium water as tritium sources, which faces drawbacks such as radioactive contamination due to the danger and instability of tritium water. Based on this, the MSD team proposed the use of metal catalyst Rh(PPh_3_)_3_Cl to activate tritium gas, and the generated metal hydride in situ was used to complete the hydrogen transfer catalysis, which realized the efficient isotope exchange of aliphatic hydrocarbon bonds and obtained tritium substitution drug molecules with high specific activity (Scheme 5b) [80].

## 4. Applications of the Tritium-Labeled Compounds

### 4.1. Cell Cytotoxicity Estimation

Evaluation of the anti-proliferative activity of drugs is one of the important methods for early drug discovery. The tritiated thymidine incorporation ([^3^H]-TdR) assay is used as an indicator of cellular proliferation and is particularly attractive for screening antimitotic agents for cancer [81,82]. Tritiated thymidine is a radiolabeled DNA precursor incorporated into newly synthesized DNA during the S-phase of the cell cycle [83,84,85]. The response value of the radioactive signal is related to the rate of proliferation, which makes it a successful agent to screen and optimize potential new drugs.

### 4.2. Human ADME Studies

The profile of a new drug’s absorption, distribution, metabolism, and excretion (ADME) properties is required for the registration, which is generally obtained in late phase I or in phase II clinical trials [86]. Preliminary metabolism data can be provided by in vitro studies. In vivo data are usually acquired by using a radiolabeled ADME study. Compared to non-radioactive techniques, such as HPLC-MS, radiolabeled compounds provide an easier and more accurate method due to their good tracer ability [87]. 

It is often the case that only when the carbon-14 option fails will the tritium-labeled compounds be used in ADME studies [88]. Many ADME scientists often carry the stereotype that tritium is a problematic isotope in terms of chemical and biological stability. However, based on the experiences and scientific data, the same ADME information can be achieved with tritium-labeled compounds as with carbon-14-labeled compounds. Along with the rapid advance in synthetic and analytic tritium chemistry and the convenient commercial reagents and equipment, the use of tritium has made major progress [2]. 

**Scheme 6:**
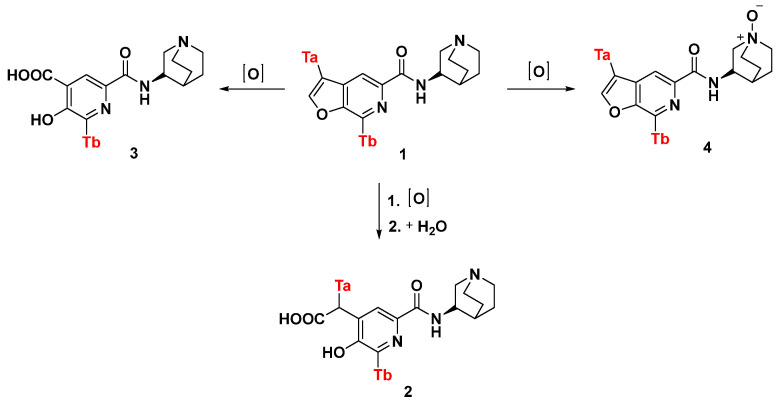
Tritium-labeled compounds in different sections (Ta and Tb) with metabolic structures that were detected analytically.

Metabolite profiling based on radioactivity monitoring of the LC eluent will provide a number of metabolites excreted via each route as well as the quantity (% of dose) or concentration (e.g., μmolEq/L). The amount of radioactivity in the analyzed sample tends to be measured using the radiological aerosol monitor (RAM) for high levels and LSC for low levels. By analyzing urine, bile, and feces, we could assess whether the drug clearance is governed by metabolism or excretion of the unchanged parent compound. Based on an agonist of the α7 nicotinic acetylcholinergic receptor, G. D. Fate et al. synthesized two tritium-labeled compounds (Scheme 6) [89]. Because of the intrinsic properties of tritium, all biological samples must undergo lyophilization to determine the content of urinary tritiated water. At the same time, using [^3^H]-**1** confirmed the species-dependent metabolism for ^3^H loss from [^3^H]-**1** in rats but not dogs. As the majority of rat metabolites resulted from furan–pyridine biotransformation, the loss of Ta was closely related to compound **3**.

The late introduction property of tritium makes it highly favored in the field of labeling complex molecules. Based on this, molecules with complex structures, such as the macrolide antibiotic [90,91], oligonucleotides [92], and polypeptides [10], have been labeled to enable pharmacokinetic, distribution, and formulation studies.

Apart from drug metabolism and pharmacokinetics studies, tritium-labeled compounds are broadly used in many other fields, such as receptor-binding studies and autoradiography.

### 4.3. Autoradiography Distribution Studies

The effects of most drugs depend on their concentrations in target tissues, while concentrations of drugs and metabolites in plasma do not directly determine those in the tissues. To this end, autoradiography distribution studies are used to provide useful information regarding tissue distribution and pharmacokinetics in drug-related material [93,94]. By using tritium-labeled drugs, the autoradiography technique can nondestructively obtain drug distributional information, which can be crucial to the studies of pharmacology, pharmacokinetics, efficacy, and toxicity [93,95]. It is worth noticing that this technique can also be used to evaluate the uptake of radioactive material in tumors [96].

**Figure 1:**
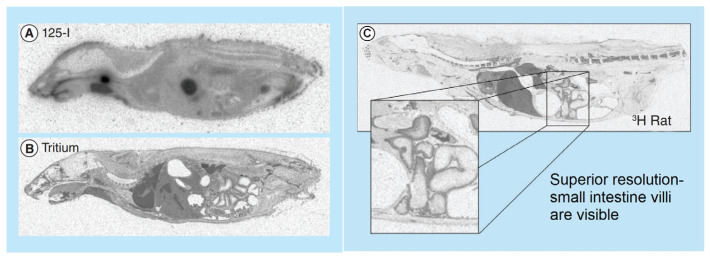
Comparison of autoradiograms obtained using iodine-125 (**A**) and tritium (**B**); representative whole-body autoradiogram obtained following administration of tritium-labeled protein to the rat (**C**) [8].

The classical radionuclide used in autoradiography distribution studies is iodine-125 [97]. However, as shown in Figure 1A, its distribution of iodine is often influenced by up-taking the free iodine to the thyroid, salivary glands, gastric mucosa, mammary glands, and ovarian follicles, resulting in an ambiguous image [8]. Fortunately, this is no issue in tritium-labeled compounds. The autoradiograms obtained from tritium have a high resolution in the images, resulting from the short path length of the beta particle emitted by tritium.

**Figure 2:**
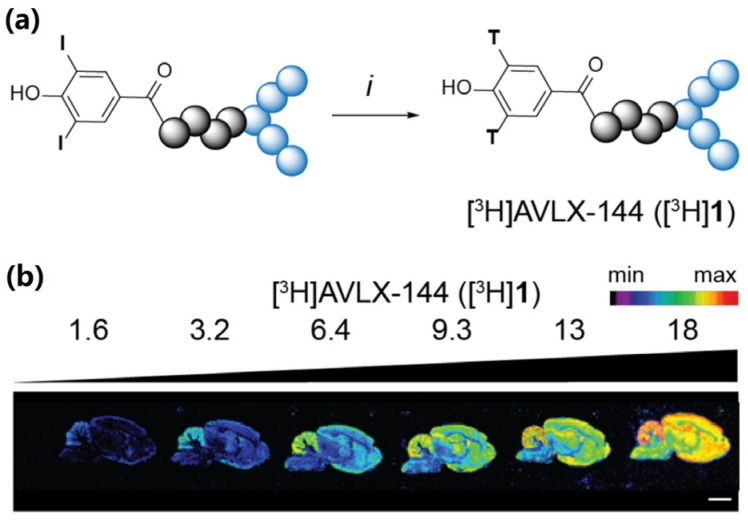
(**a**) Radiosynthesis and structure of probe [^3^H]1; (**b**) autoradiography images of sagittal rat brain slices incubated with increasing concentrations of [^3^H]1.

Recently, Kristian et al. [98] reported that probes based on the high-affinity inhibitor AVLX-144 of PSD-95 were labeled with tritium as well as fluorescent tags (Figure 2a). Tracer binding showed saturable, translocatable, and heterogeneous distributions in rat brain sections and proved effective in quantitative autoradiography and cellular imaging studies (Figure 2b).

Tritium-sucrose could be used to validate the dosing strategy in lung distribution studies of an isolated perfused mouse lung. After introducing the dose, the lung perfusion was immediately halted, and the trachea and major bronchi were isolated. All five lobes (superior, middle, inferior, post-caudal, and left) were separated using a surgical blade and separately weighed. The sample was transferred for radioactivity liquid scintillation analysis. The mass of tritium-sucrose distributed into each lobe was calculated and corrected for the lobar mass [99].

### 4.4. Tagging for Protein Binding

The high specific activity of tritium makes it a valuable tool in biological applications for detection of a variety of small molecules with their receptors. The easy detectability of the tritium also enabled the investigations of enzyme kinetics at the nanomolar level. The photoaffinity probe usually consists of three parts: a ligand with targeting properties, a photoreactive group, and a report label [100]. Compared with other identification tags, such as fluorescent dyes and affinity tags, a tritium label can minimize interference with the structure and activity of the ligand due to its small volume, thereby preserving the affinity of the ligand to the target receptor and improving the accuracy of receptor recognition [12]. In addition, the high specific activity and sensitivity of tritium can effectively reduce the background interference of non-target ligands, thus enabling in vivo activity evaluation in living cells.

**Scheme 7:**
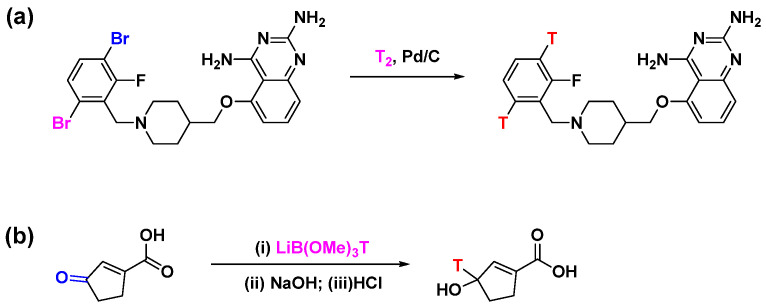
(**a**) Synthesis of tritium-labeled PF-0665247; (**b**) synthesis of tritium-labeled HOCPCA.

A tritiated chemical probe origin from a DcpS inhibitor PF-0665247 was tested against a 9000 human protein microarray to assess its binding selectivity [101]. Then, its excellent selectivity was confirmed by assessing against whole cell extracts. The tritiated probe was prepared through the tritium–halogen exchange method (Scheme 7a). Two tritium atoms were incorporated to ensure adequate specific radioactivity. High-resolution array images were received after exposure on a tritium-sensitive phosphor screen and processing through software.

To investigate the high-affinity γ-hydroxybutyrate GHB binding sites in the central nervous system, tritium-labeled 3-hydroxycyclopent-1-enecarboxylic acid (tritium-HOCPCA) was prepared using in situ generated lithium trimethoxyborotritide (Scheme 7b) [102]. The tritium incorporation approach produced an almost theoretical radioactive yield with high radiochemical purity. Based on its brain permeability, the probe is useful for understanding the molecular mechanism of GHB at its high-affinity binding site and elucidating the pharmacology of the GHB system. 

**Scheme 8:**
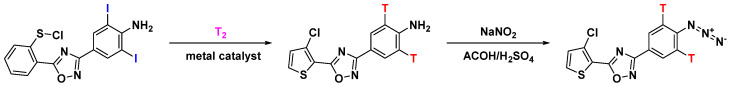
Synthesis of the tritium-labeled MX-126374 probe.

The very high but still reversible binding of probes to their receptors may be broken during the process of detection. A photoaffinity approach is commonly used to address this [103]. Photoaffinity probes replacing hydrogen atoms with tritium atoms without altering their structure will preserve the binding pattern of the drug to the maximum extent. The most extensively investigated photolabile groups are benzophenone, diazirine, and azide, among which the azide group is most widely used. By incorporating the tritium label through tritiodehalogenation, an azido- and tritium-labeled photoaffinity probe based on MX-126374 was successively synthesized (Scheme 8) [104]. Through the cell-based screening assay, a series of agents that selectively induce apoptosis in a novel pathway were found. Tail-interacting protein 47 (TIP47), a 47 kDa protein that is commonly considered a chaperone or cargo protein, is revealed as the primary target of MX-126374. The identification of the probe’s target discloses a novel and potential therapeutic selective apoptosis target.

## 5. Conclusions

Tritium labeling is an attractive field with tremendous potential for both pharmacy and chemical biology applications. With the advances in understanding of their properties, the great value of tritium-labeled compounds is emphasized in drug discovery and development. High-quality labeled compounds can be prepared rapidly and easily as a result of the rapid progress in analytical and synthesis approaches. Due to the merit of high specific activity and sensitivity, tritium-labeled compounds have been widely used in diverse fields such as human ADME studies, quantitative whole-body autoradiography, and activity-based protein profiling. We believe that their potential has not been completely exploited.

## Figures and Tables

**Table 1:** Physical properties of tritium versus those of carbon-14.

Physical Property	Tritium	Carbon-14
Half-life	12.3 years	5730 years
Specific activity	28.8 Ci/mmol	62.4 mCi/mmol
Emission type	Beta	Beta
Maximum energy of radiation	18.6 keV	156 keV
Mean energy of radiation	5.7 keV	56 keV
Decay product	^3^He^+^ (stable)	^14^N^+^ (stable)
Air	ca 6 mm	ca 20 cm
Water	ca 6 mm	ca 250 mm
Glass/concrete	ca 2 mm	ca 170 mm

## References

[1] Wang C.H., Willis D.L. (1965). Radioactive Isotopes. (Book Reviews: Radiotracer Methodology in Biological Science). Science.

[2] Uhl P., Fricker G., Haberkorn U., Mier W. (2015). Radionuclides in drug development. Drug Discov. Today.

[3] Isin E.M., Elmore C.S., Nilsson G.N., Thompson R.A., Weidolf L. (2012). Use of radiolabeled compounds in drug metabolism and pharmacokinetic studies. Chem. Res. Toxicol..

[4] Elmore C.S., Bragg R.A. (2015). Isotope chemistry; a useful tool in the drug discovery arsenal. Bioorg. Med. Chem. Lett..

[5] Marathe P.H., Shyu W.C., Humphreys W.G. (2004). The use of radiolabeled compounds for ADME studies in discovery and exploratory development. Curr. Pharm. Des..

[6] Elmore C.S. (2009). Use of Isotopically Labeled Compounds in Drug Discovery. Annu. Rep. Med. Chem..

[7] Krauser J.A. (2013). A perspective on tritium versus carbon-14: Ensuring optimal label selection in pharmaceutical research and development. J. Label. Compd. Radiopharm..

[8] McEwen A., Henson C. (2015). Quantitative whole-body autoradiography: Past, present and future. Bioanalysis.

[9] Sadaghiani A.M., Verhelst S.H., Bogyo M. (2007). Tagging and detection strategies for activity-based proteomics. Curr. Opin. Chem. Biol..

[10] Lockley W.J.S., McEwen A., Cooke R. (2012). Tritium: A coming of age for drug discovery and development ADME studies. J. Label. Compd. Radiopharm..

[11] Saljoughian M., Williams P.G. (2000). Recent developments in tritium incorporation for radiotracer studies. Curr. Pharm. Des..

[12] Atzrodt J., Derdau V., Kerr W.J., Reid M. (2018). Deuterium- and Tritium-Labelled Compounds: Applications in the Life Sciences. Angew. Chem. Int. Ed..

[13] Zouridakis N., Ochsenkuhn K.M., Savidou A. (2002). Determination of uranium and radon in potable water samples. J. Environ. Radioact..

[14] Palmblad M., Buchholz B.A., Hillegonds D.J., Vogel J.S. (2005). Neuroscience and accelerator mass spectrometry. J. Mass Spectrom..

[15] Elmore C.S., Brush K., Schou M., Palmer W., Dorff P.N., Powell M.E., Hoesch V., Hall J.E., Hudzik T., Halldin C. (2011). Synthesis of a delta opioid agonist in [^2^H_6_], [^2^H_4_], [^11^C], and [^14^C] labeled forms. J. Label. Compd. Radiopharm..

[16] Raja S.N., Surber B.W., Du J., Cross J.L. (2009). Synthesis of [^3^H] ABT-518, a matrix metalloproteinase inhibitor (MMPI) labeled in the phenyl rings. J. Label. Compd. Radiopharm..

[17] Elmore C.S., Dorff P.N., Powell M.E., Hall J.E., Simpson T.R. (2011). Synthesis of the NK3 receptor antagonist AZD2624 in C-14-, H-3-and C-13-labeled forms. J. Label. Compd. Radiopharm..

[18] Yu R.P., Hesk D., Rivera N., Pelczer I., Chirik P.J. (2016). Iron-catalysed tritiation of pharmaceuticals. Nature.

[19] Lockley W.J.S., Heys J.R. (2010). Metal-catalysed hydrogen isotope exchange labelling: A brief overview. J. Label. Compd. Radiopharm..

[20] (2010). Tritiated 2-deoxy-D-glucose: A high-resolution marker for autoradiographic localization of brain metabolism. J. Comp. Neurol..

[21] Filer C.N. (2018). Synthesis and characterization of tritium-labelled substances. Appl. Radiat. Isot..

[22] Evans E.A., Warrell D., Elvidge J., Jones J. (1985). Handbook of Tritium NMR Spectroscopy and Applications.

[23] Schenk D.J., Dormer P.G., Hesk D., Pollack S.R., Lavey C.F. (2015). NMR-based approach to the analysis of radiopharmaceuticals: Radiochemical purity, specific activity, and radioactive concentration values by proton and tritium NMR spectroscopy. J. Label. Compd. Radiopharm..

[24] Vogt F.G., Freyer A.J., Levinson S.H., Shu A.Y.L., Richard Heys J. (2005). Improved methods for ^1^H–^3^H heteronuclear shift correlation. Magn. Reson. Chem..

[25] Williams P.G., Morimoto H., Wemmer D.E. (1988). Application of modern sup ^3^H NMR techniques to analysis of complex isotopic products from a hydrogenation reaction. J. Am. Chem. Soc..

[26] Filer C.N. (1999). Isotopic fractionation of organic compounds in chromatography. J. Label. Compd. Radiopharm..

[27] Waterbeemd V.D. (1987). The parametrization of lipophilicity and other structural properties in drug design. Adv. Drug Res..

[28] Vliegen M., Janssen C., Verluyten W., Thijssen J., Cuyckens F. (2010). Tritium labelling of a new tuberculosis drug, undesired isotope effects and alternative solution via ICP-MS. J. Label. Compd. Radiopharm..

[29] Wolf J.R. (2021). Review: Self-radiolysis of compounds containing tritium and carbon-14. J. Label. Compd. Radiopharm..

[30] Eyrolle F., Ducros L., Le Dizes S., Beaugelin-Seiller K., Charmasson S., Boyer P., Cossonnet C. (2018). An updated review on tritium in the environment. J. Environ. Radioact..

[31] Ekstrom A., Garnett J.L. (1967). Recent developments in the mechanism of the protection effect of aromatic compounds on the radiolysis of aliphatics. J. Label. Compd..

[32] Sheppard G., Sheppard H.C., Stivala J.F. (1974). The stability of thymidine, uridine and their related nucleotides, labelled with tritium. J. Label. Compd..

[33] Bayly R.J., Evans E.A. (1967). Storage and stability of compounds labelled with radioisotopes—Part II. J. Label. Compd..

[34] Evans E.A., Stanford F.G. (1963). Stability of Thymidine Labelled with Tritium or Carbon-14. Nature.

[35] Browne T.R., Avenue S.H. (1997). Stable Isotopes in Pharmaceutical Research.

[36] Guengerich F.P. (2013). Kinetic deuterium isotope effects in cytochrome P450 oxidation reactions. J. Label. Compd. Radiopharm..

[37] Guroff G., Daly J.W., Jerina D.M., Renson J., Witkop B., Udenfriend S. (1967). Hydroxylation-induced migration: The NIH shift. Recent experiments reveal an unexpected and general result of enzymatic hydroxylation of aromatic compounds. Science.

[38] Sharma R., Strelevitz T.J., Gao H., Clark A.J., Schildknegt K., Obach R.S., Ripp S.L., Spracklin D.K., Tremaine L.M., Vaz A.D. (2011). Deuterium isotope effects on drug pharmacokinetics. I. System-dependent effects of specific deuteration with aldehyde oxidase cleared drugs. Drug Metab. Dispos. Biol. Fate Chem..

[39] Brues A.M., Stroud A.N., Rietz L. (1952). Toxicity of tritium oxide to mice. Proc. Soc. Exp. Biol. Med..

[40] Yang X., Liu H., Jiang X., Jin C., Xu Z., Li T., Wang Z., Wang J. (2018). Cyclooxygenase-2-mediated upregulation of heme oxygenase 1 mitigates the toxicity of deuterium-tritium fusion radiation. Int. J. Mol. Med..

[41] Arcanjo C., Adam-Guillermin C., Murat El Houdigui S., Loro G., Della-Vedova C., Cavalie I., Camilleri V., Floriani M., Gagnaire B. (2020). Effects of tritiated water on locomotion of zebrafish larvae: A new insight in tritium toxic effects on a vertebrate model species. Aquat. Toxicol..

[42] Edelmann M.R., Muser T. (2021). Tritium O-Methylation of N-Alkoxy Maleimide Derivatives as Labeling Reagents for Biomolecules. Bioconjug. Chem..

[43] Yue D., Hirao H. (2023). Mechanism of Selective Aromatic Hydroxylation in the Metabolic Transformation of Paclitaxel Catalyzed by Human CYP3A4. J. Chem. Inf. Model..

[44] Voges R., Heys J.R., Moenius T. (2009). Preparation of Compounds Labeled With Tritium and Carbon-14.

[45] Wang X.-C., Hu Y., Bonacorsi S., Hong Y., Burrell R., Yu J.-Q. (2013). Pd(II)-Catalyzed C-H Iodination Using Molecular I_2_ as the Sole Oxidant. J. Am. Chem. Soc..

[46] Allen P., Bragg R.A., Caffrey M., Ericsson C., Hickey M.J., Kingston L.P., Elmore C.S. (2017). The synthesis of a tritium, carbon-14, and stable isotope-labeled cathepsin C inhibitors. J. Label. Compd. Radiopharm..

[47] Rauh J.J., Lahm G.P., Pahutski T.F., Ullas G.V., Filer C.N. (2010). Lindlar catalyst mediated tritiation of a triazole substituted isoxazoline insecticide. J. Label. Compd..

[48] Schou S.C., Hunneche C.S., Binderup E., Grue-Sørensen G. (2005). Synthesis of tritium labelled CHS 828 and its prodrug EB 1627. J. Label. Compd. Radiopharm..

[49] Di Marco A., Cellucci A., Chaudhary A., Fonsi M., Laufer R. (2007). High-Throughput Radiometric CYP2C19 Inhibition Assay Using Tritiated (S)-Mephenytoin. Drug Metab. Dispos..

[50] Thomas S., Greenhalgh M.D. (2014). Heterogeneous Catalytic Hydrogenation of C=C and C≡C.

[51] Rylander P. (1979). Catalytic Hydrogenation in Organic Syntheses.

[52] Dutton H.J., Scholfield C.R., Selke E., Rohwedder W.K. (1968). Double bond migration, geometric isomerization, and deuterium distribution during heterogeneous catalytic deuteration of methyl oleate. J. Catal..

[53] Wyrick S.D., Susan M.N., Lauf P.K. (1984). Synthesis of tritiated bumetanide. J. Label. Compd. Radiopharm..

[54] Egan J.A., Nugent R.P., Filer C.N. (2004). Potent antiviral agent WIN 54954: High specific activity labelling with tritium. Appl. Radiat. Isot..

[55] Zhang Z., Butt N.A., Zhang W. (2016). Asymmetric Hydrogenation of Nonaromatic Cyclic Substrates. Chem. Rev..

[56] Wheeler W.J., Clodfelter D.K. (2008). Synthesis of the tritiated isotopomers of enzastaurin and its N-des-pyridylmethyl metabolite for use in ADME studies. J. Label. Compd. Radiopharm..

[57] Bolton R. (2001). Isotopic methylation. J. Label. Compd. Radiopharm..

[58] Hintermann S., Vranesic I., Allgeier H., Brülisauer A., Hoyer D., Lemaire M., Moenius T., Urwyler S., Whitebread S., Gasparini F. (2007). ABP688, a novel selective and high affinity ligand for the labeling of mGlu5 receptors: Identification, in vitro pharmacology, pharmacokinetic and biodistribution studies. Bioorg. Med. Chem..

[59] Pipal R.W., Stout K.T., Musacchio P.Z., Ren S., Graham T.J.A., Verhoog S., Gantert L., Lohith T.G., Schmitz A., Lee H.S. (2021). Metallaphotoredox aryl and alkyl radiomethylation for PET ligand discovery. Nature.

[60] Wang L., Xia Y., Derdau V., Studer A. (2021). Remote Site-Selective Radical C(sp^3^)-H Monodeuteration of Amides using D_2_O. Angew. Chem. Int. Ed..

[61] Heys J.R. (2007). Organoiridium complexes for hydrogen isotope exchange labeling. J. Label. Compd. Radiopharm..

[62] Levernier E., Tatoueix K., Garcia-Argote S., Pfeifer V., Kiesling R., Gravel E., Feuillastre S., Pieters G. (2022). Easy-to-Implement Hydrogen Isotope Exchange for the Labeling of N-Heterocycles, Alkylkamines, Benzylic Scaffolds, and Pharmaceuticals. JACS Au.

[63] Kopf S., Bourriquen F., Li W., Neumann H., Junge K., Beller M. (2022). Recent Developments for the Deuterium and Tritium Labeling of Organic Molecules. Chem. Rev..

[64] Heys R. (1992). Investigation of [IrH_2_(Me_2_CO)_2_(PPh_3_)_2_]BF_4_ as a catalyst of hydrogen isotope exchange of substrates in solution. J. Chem. Soc. Chem. Commun..

[65] Hesk D., Das P.R., Evans B. (1995). Deuteration of acetanilides and other substituted aromatics using [Ir(COD)(Cy_3_P)(Py)]PF_6_ as catalyst. J. Label. Compd..

[66] Brown J.A., Cochrane A.R., Irvine S., Kerr W.J., Mondal B., Parkinson J.A., Paterson L.C., Reid M., Tuttle T., Andersson S. (2014). The Synthesis of Highly Active Iridium(I) Complexes and their Application in Catalytic Hydrogen Isotope Exchange. Adv. Synth. Catal..

[67] Cochrane A.R., Irvine S., Kerr W.J., Reid M., Andersson S., Nilsson G.N. (2013). Application of neutral iridium(I) N-heterocyclic carbene complexes in ortho-directed hydrogen isotope exchange. J. Label. Compd. Radiopharm..

[68] Di Giuseppe A., Castarlenas R., Oro L.A. (2015). Mechanistic considerations on catalytic H/D exchange mediated by organometallic transition metal complexes. Comptes Rendus Chim..

[69] Cochrane A.R., Idziak C., Kerr W.J., Mondal B., Paterson L.C., Tuttle T., Andersson S., Nilsson G.N. (2014). Practically convenient and industrially-aligned methods for iridium-catalysed hydrogen isotope exchange processes. Org. Biomol. Chem..

[70] Daniel-Bertrand M., Garcia-Argote S., Palazzolo A., Mustieles Marin I., Fazzini P.F., Tricard S., Chaudret B., Derdau V., Feuillastre S., Pieters G. (2020). Multiple Site Hydrogen Isotope Labelling of Pharmaceuticals. Angew. Chem. Int. Ed..

[71] Stork C.M., Weck R., Valero M., Kramp H., Gussregen S., Waldvogel S.R., Sib A., Derdau V. (2023). Hydrogen Isotope Exchange by Homogeneous Iridium Catalysis in Aqueous Buffers with Deuterium or Tritium Gas. Angew. Chem. Int. Ed..

[72] Yang H., Dormer P.G., Rivera N.R., Hoover A.J. (2018). Palladium(II)-Mediated C-H Tritiation of Complex Pharmaceuticals. Angew. Chem. Int. Ed..

[73] Zhao D., Petzold R., Yan J., Muri D., Ritter T. (2021). Tritiation of aryl thianthrenium salts with a molecular palladium catalyst. Nature.

[74] Derdau V., Atzrodt J., Holla W. (2007). H/D-exchange reactions with hydride-activated catalysts. J. Label. Compd. Radiopharm..

[75] Derdau V., Atzrodt J. (2006). C-H/C-D Exchange Reactions of Aromatic Compounds in D_2_O with NaBD_4_—Activated Catalysts. Synlett.

[76] Martinez-Prieto L.M., Chaudret B. (2018). Organometallic Ruthenium Nanoparticles: Synthesis, Surface Chemistry, and Insights into Ligand Coordination. Acc. Chem. Res..

[77] Derdau V., Elmore C.S., Hartung T., McKillican B., Mejuch T., Rosenbaum C., Wiebe C. (2023). The Future of (Radio)-Labeled Compounds in Research and Development within the Life Science Industry. Angew. Chem. Int. Ed..

[78] Lepron M., Daniel-Bertrand M., Mencia G., Chaudret B., Feuillastre S., Pieters G. (2021). Nanocatalyzed Hydrogen Isotope Exchange. Acc. Chem. Res..

[79] Loh Y.Y., Nagao K., Hoover A.J., Hesk D., Rivera N.R., Colletti S.L., Davies I.W., MacMillan D.W.C. (2017). Photoredox-catalyzed deuteration and tritiation of pharmaceutical compounds. Science.

[80] Yang H., Huang Z., Lehnherr D., Lam Y.H., Ren S., Strotman N.A. (2022). Efficient Aliphatic Hydrogen-Isotope Exchange with Tritium Gas through the Merger of Photoredox and Hydrogenation Catalysts. J. Am. Chem. Soc..

[81] Goodlad R.A. (2017). Quantification of epithelial cell proliferation, cell dynamics, and cell kinetics in vivo. Wiley Interdiscip. Rev. Dev. Biol..

[82] Griffiths M., Sundaram H. (2011). Drug design and testing: Profiling of antiproliferative agents for cancer therapy using a cell-based methyl-[3H]-thymidine incorporation assay. Cancer Cell Cult. Methods Protoc..

[83] Mewissen D.J., Furedi M., Ugarte A., Rust J.H. (1978). Comparative incorporation of tritium from tritiated water versus tritiated thymidine, uridine or leucine. Curr. Top. Radiat. Res. Q..

[84] Nagao S., Ikegami S., Tanaka A. (1984). Inhibition of macrophage DNA synthesis by immunomodulators. II. Characterization of the suppression by muramyl dipeptide or lipopolysaccharide [^3^H]thymidine incorporation into macrophages. Cell. Immunol..

[85] Prasad H.K., Nath I. (1981). Incorporation of 3H-thymidine in Mycobacterium leprae within differentiated human macrophages. J. Med. Microbiol..

[86] Liu Y., Hong L., Yu L.S., Jiang H.D., Chen J.Z., Meng Q., Chen S.Q., Zeng S. (2011). The role of ADME evaluation in translation research of innovative drug. Yao Xue Xue Bao.

[87] Goodman K.J., Brenna J.T. (1992). High sensitivity tracer detection using high-precision gas chromatography-combustion isotope ratio mass spectrometry and highly enriched [U-13C]-labeled precursors. Anal. Chem..

[88] Loewe C., Atzrodt J., Reschke K., Schofield J. (2016). Conception, realization and qualification of a radioactive clean room lab facility dedicated to the synthesis of radiolabeled API for human ADME studies. J. Label. Compd. Radiopharm..

[89] Shaffer C.L., Gunduz M., Thornburgh B.A., Fate G.D. (2006). Using a tritiated compound to elucidate its preclinical metabolic and excretory pathways in vivo: Exploring tritium exchange risk. Drug Metab. Dispos..

[90] Sutherland I.H., Campbell W.C. (1990). Development, pharmacokinetics and mode of action of ivermectin. Acta Leiden..

[91] Rotert G., Fan L., Shevchenko V.P., Nagaev I.Y., Myasoedov N.F., Susan A.B. (2006). Syntheses of radiolabeled ABT-578 for ADME studies. J. Label. Compd. Radiopharm..

[92] Sands H., Gorey-Feret L.J., Ho S.P., Bao Y., Cocuzza A.J., Chidester D., Hobbs F.W. (1995). Biodistribution and metabolism of internally 3H-labeled oligonucleotides. II. 3′,5′-blocked oligonucleotides. Mol. Pharmacol..

[93] Solon E.G. (2007). Autoradiography: High-resolution molecular imaging in pharmaceutical discovery and development. Expert Opin. Drug Discov..

[94] Solon E.G. (2015). Autoradiography techniques and quantification of drug distribution. Cell Tissue Res..

[95] Stumpf W.E. (2005). Drug localization and targeting with receptor microscopic autoradiography. J. Pharmacol. Toxicol. Methods.

[96] Ciprotti M., Chong G., Gan H.K., Chan A., Murone C., Macgregor D., Lee F.T., Johns T.G., Heath J.K., Ernst M. (2014). Quantitative intratumoural microdistribution and kinetics of ^131^I-huA33 antibody in patients with colorectal carcinoma. EJNMMI Res..

[97] Dewanjee M.K. (1992). Radioiodination: Theory, Practice, and Biomedical Applications.

[98] Fernandes E.F.A., Palner M., Raval N.R., Jeppesen T.E., Dankova D., Baerentzen S.L., Werner C., Eilts J., Maric H.M., Doose S. (2024). Development of Peptide-Based Probes for Molecular Imaging of the Postsynaptic Density in the Brain. J. Med. Chem..

[99] Al-Jayyoussi G., Price D.F., Francombe D., Taylor G., Smith M.W., Morris C., Edwards C.D., Eddershaw P., Gumbleton M. (2013). Selectivity in the impact of P-glycoprotein upon pulmonary absorption of airway-dosed substrates: A study in ex vivo lung models using chemical inhibition and genetic knockout. J. Pharm. Sci..

[100] Panov M.S., Voskresenska V.D., Ryazantsev M.N., Tarnovsky A.N., Wilson R.M. (2013). 5-Azido-2-aminopyridine, a new nitrene/nitrenium ion photoaffinity labeling agent that exhibits reversible intersystem crossing between singlet and triplet nitrenes. J. Am. Chem. Soc..

[101] Hett E.C., Kyne R.E., Gopalsamy A., Tones M.A., Xu H., Thio G.L., Nolan E., Jones L.H. (2016). Selectivity determination of a small molecule chemical probe using protein microarray and affinity capture techniques. ACS Comb. Sci..

[102] Vogensen S.B., Marek A., Bay T., Wellendorph P., Kehler J., Bundgaard C., Frølund B., Pedersen M.H.F., Clausen R.P. (2013). New Synthesis and Tritium Labeling of a Selective Ligand for Studying High-Affinity γ-Hydroxybutyrate (GHB) Binding Sites. J. Med. Chem..

[103] Anabuki T., Tsukahara M., Matsuura H., Takahashi K. (2016). Tandem photoaffinity labeling of a target protein using a linker with biotin, alkyne and benzophenone groups and a bioactive small molecule with an azide group. Biosci. Biotechnol. Biochem..

[104] Jessen K.A., English N.M., Wang J.Y., Maliartchouk S., Drewe J., Kuemmerle J., Zhang H.Z., Tseng B., Cai S.X., Kasibhatla S. (2005). The discovery and mechanism of action of novel tumor-selective and apoptosis-inducing 3,5-diaryl-1,2,4-oxadiazole series using a chemical genetics approach. Mol Cancer Ther..

